# The Role of Virtual Reality in Screening, Diagnosing, and Rehabilitating Spatial Memory Deficits

**DOI:** 10.3389/fnhum.2021.628818

**Published:** 2021-02-05

**Authors:** Miles Jonson, Sinziana Avramescu, Derek Chen, Fahad Alam

**Affiliations:** ^1^School of Medicine, College of Health and Agricultural Sciences, University College Dublin, Dublin, Ireland; ^2^Department of Anesthesia, Sunnybrook Health Sciences Centre, Toronto, ON, Canada; ^3^Department of Anesthesia and Pain Medicine, Sunnybrook Health Sciences Centre, University of Toronto, Toronto, ON, Canada; ^4^Department of Anesthesia, Humber River Hospital, Toronto, ON, Canada; ^5^School of Pharmacy, University of Waterloo, Waterloo, ON, Canada

**Keywords:** spatial memory, virtual reality, Alzheimer’s disease, mild cognitive impairment, cognitive assessment, rehabilitation

## Abstract

Impairment of spatial memory, including an inability to recall previous locations and navigate the world, is often one of the first signs of functional disability on the road to cognitive impairment. While there are many screening and diagnostic tools which attempt to measure spatial memory ability, they are often not representative of real-life situations and can therefore lack applicability. One potential solution to this problem involves the use of virtual reality (VR), which immerses individuals in a virtually-simulated environment, allowing for scenarios more representative of real-life without any of the associated risks. Here, we review the evidence surrounding the use of VR for the screening and diagnosis of spatial memory impairments, including potential limitations and how it compares to standard neuropsychological tests. We will also discuss the evidence regarding the potential use of VR in the rehabilitation of spatial memory deficits, which has not been well studied, but which could be game-changing if proven successful.

## Introduction

Virtual Reality (VR) is a promising modality for the screening, diagnosis, and possible rehabilitation of mild cognitive impairment (MCI) and Alzheimer’s disease (AD). A VR task can range in its presentation from non-immersive to immersive VR (D’Cunha et al., 2019). Non-immersive VR involves the projection of the virtual environment from a 2D screen, analogous to watching television. Immersive VR uses head-mounted devices to immerse users in a 3D environment; this offers 360° audio and visual stimuli, allowing users to experience a greater sense of presence within the computer-simulated 3D environment (D’Cunha et al., 2019). Presence is the depth to which a participant feels that they are submerged within a simulated world. In comparison to non-immersive VR, immersive VR leverages the vestibular and proprioceptive systems to further engage the participant and increase spatial memory recall, an important marker for MCI and AD (Krokos et al., 2019). To enhance the sensation of presence, reality can be designed to mimic authentic situations requiring the use of spatial memory (van der Ham et al., 2015). For example, Davis et al. (2017) modeled their VR on a real senior residential community, which their participants navigated daily. The inherent safety of VR allows patients to participate in potentially dangerous tasks, such as navigating an area or driving a car, from the comfort of a rigorously controlled testing environment (Maguire et al., 2006; Plancher et al., 2012). Spatial memory is an important part of navigating these types of challenges and impairment is often the first sign of functional disability in the course of MCI and AD (Burgess et al., 2002; Coughlan et al., 2018). This cognitive process is broad and involves recalling details of previous locations, finding objects, and navigating the world (Astur et al., 2002). Deficits can lead to wandering and life-threatening driving collisions, which can result in daily stresses, the loss of independent living, and, not least of all, harm to the patient or those around them (Cushman et al., 2008).

As an early symptom of MCI and AD, spatial memory may be a useful marker of cognitive impairment (Coughlan et al., 2018). There are several potential benefits to a timely diagnosis of MCI and AD, not just for patients, but for caregivers and society, too (Dubois et al., 2015). For example, early intervention can improve the quality of life, allow early access to support services, enable planning for the future (Boise et al., 1999; De Vugt and Verhey, 2013), and hopefully, in the future, provide an opportunity for early management with disease-modifying therapies, which as of yet are unavailable. Advancing the diagnostic capabilities of spatial memory assessments may therefore allow for earlier administration of disease-modifying therapies which, if effective, may improve the safety, quality of life, and decrease the morbidity and mortality of patients diagnosed with MCI and early-onset AD (Coughlan et al., 2018).

Many of the current cognitive screening tools have been criticized for their poor sensitivity and specificity, particularly with regards to differentiating MCI from healthy aging (De Roeck et al., 2019). Some of these tests are very short to administer (<5 min) and they are used to screen for cognitive impairment, while others are longer duration tests (6–20 min), normally administered in more specialized settings, and can be used longitudinally for a follow-up to assess the effect of potential interventions or the progression of the disease. Of the 50 screening tools for MCI and AD reviewed by De Roeck et al. (2019), only 20 contained a visuospatial component, such as the *Clock Drawing Test*, the *Cube Drawing Test*, and the *Intersecting Shapes Test*.

Despite the portion of the current screening and diagnostic assessments which do measure a component of spatial memory, most do not adequately measure an individual’s spatial memory in scenarios representative of real-life situations (De Roeck et al., 2019). Evidence in this review supports the idea that both non-immersive and immersive VR are more sensitive and ecologically valid than standard neuropsychological tests in the assessment of MCI and AD. Ecological validity refers to the degree to which the findings of a study or a diagnostic assessment relate to a real-world setting; in this case, how well the VR spatial memory task represents real-life spatial memory challenges faced by MCI and AD patients. Studies have proven the utility of assessing spatial memory in both real and VR large-scale environments (Cushman et al., 2008; Cogné et al., 2017; Zhou et al., 2020). Several publications have shown a correlation in performance on spatial memory tasks between real and virtual environments (Hort et al., 2007; Cushman et al., 2008; van der Ham et al., 2015). Patient performance on tasks within a virtual environment may therefore be a stronger indicator of their future performance on related tasks of daily living than the current neuropsychological assessments available.

Following a diagnosis of spatial memory impairment, patients have limited rehabilitation options available to them. As well as having a screening and diagnostic value, VR has the potential to be used as a rehabilitation tool for these patients (Weniger et al., 2011; Caglio et al., 2012; Kober et al., 2013; Guderian et al., 2015; Claessen et al., 2016; Montana et al., 2019). If shown to be effective, VR rehabilitation could improve patients’ capacity to pursue their personal interests by resolving the challenges of living with spatial memory impairment. Currently, strategies involving VR have not been able to elicit comprehensive functional rehabilitation and this continues to be an area of interest for future studies.

This review article will discuss: (1) the utility of VR for the screening and diagnosis of spatial memory impairments; (2) the role of VR in the rehabilitation of spatial memory deficits; (3) the limitations of VR tests; and (4) the advantages and disadvantages of different design options for VR-based tests and rehabilitation tools.

## Screening and Diagnosis of Spatial Memory Impairment Using VR

The utility of VR for screening and diagnosis of spatial memory deficits is based on its ability to: (i) mimic real-world tasks; (ii) discriminate between healthy controls (HCs) and patients with cognitive impairment; and (iii) compete in efficacy with standard neuropsychological tests.

### Ecological Validity: The Ability to Mimic Real-World Tasks

Similarly formatted real-life and VR assessments were compared to assess ecological validity (Maguire et al., 2006; Hort et al., 2007; Cushman et al., 2008; van der Ham et al., 2015; Cogné et al., 2017). The comparisons discussed below show a correlation between spatial memory assessed in both physical environments, such as in a university building, a hospital, and a Morris Water Maze, as well as in VR versions of the same environments. Cushman et al. (2008) compared participants’ ability to learn a route through a hospital with their ability to learn a virtual analog of the same route. While it should be noted that scores on the eight subtests measuring *route learning*, *free recall of objects*, *self-orientation*, *route drawing*, *landmark recall*, *photograph recognition*, *photograph location*, and *video location* were slightly lower on the non-immersive VR component than those on its real-world counterpart, both were able to differentiate between HCs and MCI patients (Cushman et al., 2008).

Hort et al. (2007) demonstrated a similar ability to diagnose spatial memory impairment with both a physical version of the Morris Water Maze and a nearly identical non-immersive VR counterpart. The Morris Water Maze assesses a patient’s spatial memory by having them repeatedly locate a hidden goal from memory in either a physical or VR environment (Vorhees and Williams, 2014). The task tests the participants’ ability to remember the location of something, without having seen it placed there immediately before. This is similar to leaving an object concealed in a room and then returning to the room to retrieve it. Unfortunately, while the results of this comparative study showed an effective transferability between a real and VR Morris Water Maze, it is not a great representation of the real-world. The controllability, ease of use, and lack of distractions may simplify the challenges of a similar real-world task, which detracts from its ecological validity. Furthermore, Learmonth et al. (2008) argue that a small virtual world may be a poor representation of real-world navigation.

van der Ham et al. (2015) compared the navigational abilities of healthy participants in a real university building, a VR version, and a real/VR hybrid. The participants either walked through a university building, traveled through a classic non-immersive VR, or walked a path on an outdoor field while looking at a Samsung Galaxy Tab 1, which was displaying an image of the non-immersive VR in real-time as the participant walked where the building would be (a type of semi-immersive VR). Spatial memory was assessed by the following subtests: *landmark recognition*, *estimated route distance*, the *position of an object*, *pointing task (direction towards start and end from random position)*, and *map drawing*. Performance in the *pointing task* and *map drawing* was shown to be significantly higher in the real and hybrid tasks compared with the virtual task but *landmark recognition*, *estimated route distance*, and *position of an object* were all the same. This may indicate that certain elements of spatial memory are more strongly stimulated by physically traveling through space (van der Ham et al., 2015).

Deeper immersion may increase the ecological validity of VR spatial memory assessments. Immersive VR tasks can be attempted without subjecting the patient to the tangible risks associated with AD. For example, driving a vehicle through immersive virtual cities, such as London or Paris (Maguire et al., 2006; Plancher et al., 2012) uses a participant’s vestibular and proprioceptive systems to increase spatial memory recall (Krokos et al., 2019), while mitigating the risks associated with operating a vehicle. We believe the increased sense of presence within the virtual environment, provided by immersive VR, will improve an assessment’s applicability to the real world, thus improving its ecological validity. Despite this, several cognitive processes necessary for driving are vulnerable to age-related decline. These include declines in executive function, working memory, attention, and speed of information processing (Miller et al., 2016). This may result in difficulty separating spatial memory from these other cognitive processes.

Other ecologically valid VR formats include the town walkthrough, park exploration, senior residential community, cycle through a city and grocery shop (Ferrara et al., 2008.; van der Ham et al., 2010; Weniger et al., 2011; Davis et al., 2017; Mrakic-Sposta et al., 2018). Environments may be entirely fictional representations or more specific. For example, Ferrara et al. (2008) had participants walk through a generic simulated town, while van der Ham et al. (2010) had participants walk through a VR version of the real German town, Tubingen. Weniger et al. (2011) had participants explore a non-immersive virtual park with the task of finding a hidden goal. Davis et al. (2017) used a VR analog of a real senior residential community providing an environment that is thoroughly representative of the daily experience of its users. Mrakic-Sposta et al. (2018) had participants use a stationary bike attached to a non-immersive VR display, to simulate cycling through a city, followed by simulated shopping at a grocery store. Multiple activities tested in unison by Mrakic-Sposta et al. (2018) simulates the multiple spatial tasks a person may need to undertake to complete a single goal.

### Ability to Discriminate Between Individuals With Cognitive Impairment and HCs

Early detection of MCI and AD is vital for the current treatment strategies of counseling, psycho-education, cognitive training, and medication to provide effective intervention (De Roeck et al., 2019). VR may act as a screening and diagnostic tool for MCI and AD (Cushman et al., 2008; Weniger et al., 2011; Plancher et al., 2012; Davis et al., 2017; Montenegro and Argyriou, 2017) if it can discriminate first between HC and MCI, and second between MCI and AD. Indeed, VR assessments in current literature have shown success in discriminating between groups of HCs, MCI patients, and AD patients (Cushman et al., 2008; Weniger et al., 2011; Plancher et al., 2012). MCI is regarded as a prodromal transition stage that presents before the development of fully symptomatic AD and can be difficult to diagnose as it is a heterogeneous condition with multiple phenotypes of cognitive function loss (Petersen et al., 2006; D’Cunha et al., 2019; De Roeck et al., 2019). MCI may be subtyped as amnestic, in the case of memory impairment, or non-amnestic, in the case of attention, language, or visuospatial impairment. MCI can be further stratified into a single domain or multiple domains depending on the number of cognitive systems affected (Roberts and Knopman, 2013). The subtypes are important to separate from one another as different presentations are more frequently associated with specific diseases. For example, neurodegenerative diseases, such as AD, are more likely to be the cause of amnestic MCI and present with memory impairment, while non-amnestic MCI is more likely to develop from cerebrovascular disease and present in one of the remaining domains (Roberts and Knopman, 2013). It is important to identify MCI patients as well as AD patients to ensure early management of the disease takes place.

In two non-immersive VR tasks assessing spatial memory, MCI patients demonstrated significantly more navigation errors than HCs (Weniger et al., 2011). The first task involved exploring a virtual park towards an end-goal in an unknown location. Mistakes, on subsequent attempts to reach the same goal, such as visiting incorrect locations, were measured. Throughout five attempts both MCI patients and HCs showed significant improvement, however, at each trial, the MCI patients performed more errors than HCs (Weniger et al., 2011). The second task had participants navigate a virtual maze and, similarly, over five attempts their progress and mistakes were measured. The task showed significant improvement in performance by HCs, but not in participants with MCI (Weniger et al., 2011).

Identifying the severity of impairment is crucial in determining clinical outcomes and treatment strategies for patients with MCI and AD (De Roeck et al., 2019). Therefore, differentiating between the two states is important for achieving the highest standard of care for patients. Plancher et al. (2012) showed that on memory tasks, such as recalling the locations of landmarks after driving through a non-immersive VR simulation, AD patients performed significantly worse than MCI patients, who performed significantly worse than HCs. The three participant groups were distinguishable from one another in subtests measuring recall of what was seen, surrounding details, associated times, egocentric position, and allocentric position (Plancher et al., 2012). Nearly identical group trends were observed in the Cushman et al. (2008) study using non-immersive VR to measure various components of spatial memory. Scores on *navigation* and *object location accuracy* were the best predictors of participants’ group status, which is in line with the belief that these tasks require spatial memory (Cushman et al., 2008). Hort et al. (2007) observed similar trends in performance with progression towards AD on their non-immersive VR test. They had participants (HCs, non-amnestic MCI patients, amnestic MCI patients, multiple domain MCI patients with an amnestic component, and AD patients) complete both a real and VR Morris Water Maze (Hort et al., 2007). Comparison of the results between groups showed a steady decline in performance proportional to the level of cognitive impairment.

Recent data suggest that, like non-immersive VR, immersive VR can differentiate AD patients from HCs (Montenegro and Argyriou, 2017). In an immersive VR study, Montenegro and Argyriou (2017) measured AD patients’ ability to accurately recall the positions of objects in a virtual room and observed that AD patients scored significantly lower than HCs. The average percentage of objects recalled by AD patients was 23.8% compared with 92.5% by HCs (Montenegro and Argyriou, 2017). This indicates that immersive VR, like non-immersive, can detect spatial memory deficits and can differentiate between HCs and AD, which suggests the diagnostic potential for these devices. Montenegro and Argyriou (2017) did not include MCI patients in their study.

Due to the well documented cognitive and personality changes associated with AD, differentiating between healthy aging and AD is a more straightforward task than separating healthy aging from MCI. The following section addresses how VR assessments may be superior to current neuropsychological assessments in this regard.

### VR Testing: Comparison to Standard Neuropsychological Tests

Despite the wide variety of screening instruments available, due to the lack of sensitivity and specificity, it is not clear which assessment is best suited for early detection of AD (De Roeck et al., 2019). The reliability of a given test for predicting MCI and AD seems to depend on its ecological validity (Lesk et al., 2014). More practical and involved tasks, relying on the visual and vestibular systems, may increase the participants’ use of spatial memory systems (Krokos et al., 2019), circumventing the limited ecological validity of the current neuropsychological tests (Chua et al., 2019). VR modalities may therefore result in improved diagnostic accuracy compared with the standard neuropsychological tests, including the Mini Mental-State Examination (MMSE; Arevalo-Rodriguez et al., 2015), the Wechsler’s Memory Scale (WMS; Brooks et al., 2008), and the Rivermead Behavioural Memory Test—3rd Edition (RBMT-3; Weniger et al., 2011).

The MMSE screening tool assesses general cognitive functions with a set of 11 questions. Spatial perception is assessed by the participant’s ability to copy two overlapping figures (Arevalo-Rodriguez et al., 2015), which may not be wholly representative of spatial challenges faced in real-life, such as navigation or locating household objects. Spatial memory is further assessed by recalling the patient’s location starting with the building they are currently in and working through their district, town, and state. Multiple studies have observed that the MMSE scores of HCs, MCI, and AD patients vary significantly (Cushman et al., 2008; Caffò et al., 2012; Plancher et al., 2012). However, when separating HCs from MCI patients, contradicting evidence suggests that the MMSE lacks statistical power as a diagnostic tool (Mitchell, 2009; Guo et al., 2012; Lesk et al., 2014). The estimated correct classification rate of the MMSE was 95% between HCs and AD patients, but under 70% between HCs and MCI patients (Guo et al., 2012). Comparatively, using a non-immersive virtual Morris Water Maze, Caffò et al. (2012) correctly classified over 85% of HCs and MCI patients. Lesk et al. (2014) similarly showed that VR modalities measured statistically significant variation between HCs and MCI patients, and achieved group classification more strongly correlated with the presence of MCI associated brain pathology than that seen with the MMSE. Thus, although there are no studies that directly compare the two, there is some evidence that VR may be superior to the MMSE for the screening and diagnosis of MCI.

Similar observations were made for the WMS, a general memory test (Weniger et al., 2011). The spatial memory component of the WMS-R (revised edition) involves recall and recognition of basic designs (Weniger et al., 2011). Multiple studies have reported that HCs and MCI patients score sufficiently differently on the WMS to confirm the diagnosis of MCI (Weniger et al., 2011; Guderian et al., 2015). However, one analysis concluded that the measurement of multiple memory domains by the more recent WMS-3 (3rd edition) would lead to 26% of healthy older adults scoring low on at least one domain, increasing their likelihood of being misclassified as having MCI (Brooks et al., 2008). Guderian et al. (2015) noted that WMS-3 scores were unable to explain all of the spatial memory deficits measured using their own VR spatial memory assessment.

Possibly the most ecologically valid of the standard tests of spatial memory is the RBMT-3, which assesses general memory using a series of subtests (Weniger et al., 2011). For the spatial memory component of the RBMT-3 (subtest: *route recall*), participants are asked to retrace the walking route of an examiner within a room (Weniger et al., 2011). Weniger et al. (2011) found that MCI patients and HCs scored significantly differently on the RBMT. This subtest is similar to realistic navigational challenges. However, it is not conducted in a standardized environment and therefore results are subject to variability based on the specific environment and examiner. Conversely, virtual environments are highly controlled, with every detail programmed and predetermined, and can easily be shared between sites, making VR a more consistent and reproducible diagnostic tool (van der Ham et al., 2015). The improved replicability and removal of examiner variability achieved with VR-based tests may result in increased sensitivity and specificity in assessments of MCI and AD.

## Spatial Memory Rehabilitation

Rehabilitation of spatial memory using VR shows mixed results. In a systematic literature review of 16 VR rehabilitation studies, Montana et al. (2019) argue that the current literature shows positive results regarding VR spatial memory rehabilitation and that the most important characteristics indicating success are the overall duration of the training, the frequency, the intensity of each session, and the time elapsed since damage to spatial centers was sustained. On the other hand, an inspection of some of the studies seems to indicate that rather than genuine rehabilitation of spatial memory function, a learning effect may take place (Brooks, 1999; Hofmann et al., 2003; Claessen et al., 2016; Montana et al., 2019). A learning effect occurs when there is an apparent improvement in the ability to reproduce a route, procedure, or skill following exposure and repetition, however, upon alteration of the task, for example by introduction of a new or slightly altered route, the patient is no faster or more able to complete the task than they were before they had undergone rehabilitation. This improvement in performance on a single task without transference to generalized cognitive improvement is a very difficult hurdle to overcome in brain training technology (Owen et al., 2010; Stojanoski et al., 2020). Thus, the improvement of capacity to complete novel tasks may be a more accurate indication of spatial memory rehabilitation.

Claessen et al. (2016) studied the rehabilitation of six stroke patients using non-immersive VR. Patients’ spatial navigation abilities were assessed using the Virtual Tubingen test, which shows a video of a virtual route through the German city Tubingen and then asks patients to complete 10 subtests (*scene recognition, route continuation, route sequence, route order, route progression, route distance, pointing to start, pointing to end, map drawing, and map recognition*) to determine route memorization and survey knowledge of the route. Based on the weaknesses assessed by the Virtual Tubingen test, Claessen et al. (2016) created a tailored learning approach to supplement each of the participants’ needs. Patients using mostly route memorization were given survey-based tasks, such as using the shapes of buildings, to learn their route, while patients using mostly survey-based memorization were given route memorization tasks, such as planning the shortest route on a map and using a birds-eye-view to supplement navigation. Patients then had three remaining hour-long sessions dedicated to improving their navigation skills in the non-immersive VR of Tubingen. Five of the six patients showed improvement in the navigation skill they were intentionally training (Claessen et al., 2016). However, five of the six also showed negative effects on many of the other navigation abilities, which were not trained (Claessen et al., 2016). For example, one participant prepared a map of the route using survey-based techniques and improved in the *route progression* and *route distance* subtests, while they markedly decreased in scores on the *pointing tasks*. This seems to suggest skill learning took place, as instead of gross spatial memory improvement, only the specific skill worked on was improved.

For 4 weeks, Hofmann et al. (2003) investigated participants’ spatial memory by having them travel through a virtual town, collecting items along the way. At key navigational decision moments, participants were asked multiple-choice questions related to the decision they made (for example: what is the name of a building in the landscape?) to stimulate spatial memory formation (Hofmann et al., 2003). Participants showed a slight improvement over successive VR trials, however no improvement in neuropsychological testing was found (Hofmann et al., 2003). When comparing the results in training periods and test trials, Hofmann et al. (2003) determined that they had measured a procedural memory learning effect. Participants improved their ability to perform a specific task rather than rehabilitated their spatial memory deficits. This may be attested to the task of navigating a strict route and using a touch screen to operate the movements, resulting in highly repetitive motor movements. Analysis of MMSE scores taken before and after the experiment showed no improvement in AD patients, while a 1-point mean increase was noted in HCs (Hofmann et al., 2003). In future studies, it will be important to make the distinction between the assessment of skill learning and the assessment of memory forming capacity, to avoid misleading conclusions. More recent performance improvements on neuropsychological spatial memory assessments following VR rehabilitation have shown that some spatial memory restoration may be possible (Caglio et al., 2012; Kober et al., 2013).

As the primary purpose of rehabilitation is to establish long-term functional memory, Caglio et al. (2012) looked at improvements in spatial memory in both the short and long term following an extensive non-immersive VR rehabilitation program undertaken by a single patient with a traumatic brain injury. In this study, the patient was instructed to explore a virtual city while cutting down trees and telephone poles, with the implicit spatial memory task of avoiding previously explored routes. This spatial memory training program included three 90-min sessions per week for 5 weeks. The patient spent 22.5 h in VR rehabilitation and Montana et al. (2019) in part, characterized this study’s success by its overall duration. Improvement in spatial memory was measured before and after the program using eight neuropsychological assessments and showed significant improvement in the RBMT delayed route recall subtest. After the rehabilitation program, scores on the delayed route recall increased from 0 to 80% correct, and this improvement was maintained in the 2-month follow-up. In the 1-year follow-up, the patient scored 100% correct. Also, functional magnetic resonance imaging revealed increased activation in many brain structures, including the left and right hippocampi (Caglio et al., 2012). The hippocampi, often damaged in MCI and AD, are associated with spatial memory formation and storage (Burgess et al., 2002; Weniger et al., 2011) and hippocampal volume is strongly associated with spatial memory functioning (Schuff et al., 2009). Therefore, increased activation of the hippocampi may indicate successful rehabilitation of spatial memory. Despite this apparent success, the nature of a single patient study limits the ability of an interpreter to make statements regarding its efficacy.

Kober et al. (2013) investigated the short-term rehabilitation effects of VR training on eleven patients with severe spatial memory deficits. They administered various neuropsychological tests, including the Benton Test, the Achievement Measure System 50+, the Visual Pursuit Test, and the Corsi Block-Tapping Test, followed by five non-immersive VR navigation tasks and then repeated the same neuropsychological battery (Kober et al., 2013). Participants completed up to three route learning simulations per 20-min session, in which they were directed through a virtual city and attempted to complete the routes from memory. Each route was repeated until it was learned to completion and a score was quantified based on the number of mistakes made and the number of routes learned (Kober et al., 2013). Similarly to Hofmann et al. (2003), they showed that patients improved during successive VR trials, but conversely, they found that patients also improved slightly on three out of the four neuropsychological assessments (Kober et al., 2013). MCI patients significantly improved their scores on the Benton Test, the Achievement Measure System 50+, and the Visual Pursuit Test, while, interestingly, HCs only improved their performance on the Benton Test (Kober et al., 2013). The Benton test measures visual perception and visual memory. It is unclear why Kober et al. (2013) showed improvement on neuropsychological testing in contrast with the results of Hofmann et al. (2003), however, the results indicate the possibility of increased cognitive function following VR intervention. Although these results are promising, Kober et al. (2013) do not comment on long-term rehabilitation or how the participants’ spatial memory might have improved in tasks related to daily living.

Mrakic-Sposta et al. (2018) showed promising results with their immersive VR rehabilitation. Ten mild to moderate MCI patients were given a pre-and post-VR neuropsychological battery of tests including visuospatial function assessment with the MMSE, the Rey-Osterrieth Complex Figure Test, and the Attentional Matrices Test. The Rey-Osterrieth Complex Figure Test measures the patients’ degree of constructional apraxia by having them draw a copy of a geometric picture. The Attentional Matrices Test monitors visual attention and memory by having patients delete numbers that match target numbers in an array of distractors. Half of the patients were assigned to be in the experimental group, while the other half were treated as controls. The experimental group had three sessions per week for 6 weeks, while the control group had none. During the sessions, the experimental group performed three VR tasks including riding a stationary bike through a park (stationary bike was connected to the VR system to simulate movement), avoiding cars while cycling through a city, and finally shopping for groceries in a supermarket. This three-tiered VR demonstrates good ecological validity as it demonstrates the multi-task nature of daily living. Assessment of pre-and post- VR scores on neuropsychological tests showed improvement of the experimental group in the MMSE, the Rey-Osterrieth Complex Figure Test, and the Attentional Matrices Test compared with the control group who worsened in all tests. Additionally, a marginal increase in the experimental group’s scores on the Functional Activity Questionnaire showed a slight improvement in the patient’s independence regarding activities of daily living. Mrakic-Sposta et al. (2018) noted the small patient population as a limitation to the validity of their results.

Spatial memory rehabilitation using VR has not been researched extensively (Caglio et al., 2012; Kober et al., 2013; Mrakic-Sposta et al., 2018). Instead, VR rehabilitation studies on MCI and AD have more often demonstrated the preservation of skill learning capacity or procedural memory formation *via* the repetition of specific tasks (Brooks, 1999; Moffat et al., 2001; Hofmann et al., 2003; Weniger et al., 2011; Lesk et al., 2014; Claessen et al., 2016). Because the skills learned in a particular task are not generalized, they have not been shown to cross brain training platforms and this remains a common limitation of neuropsychological rehabilitation (Owen et al., 2010; Stojanoski et al., 2020). However, there is an opportunity for future studies to focus on the long-term rehabilitation of spatial memory deficits that can be extrapolated to unrelated tasks.

## Limitations of VR Spatial Memory Assessments

As with any new testing modality, VR should be vigorously vetted for its utility in a clinical setting. While the ability of VR to make accurate assessments of spatial memory deficits is supported by the literature, it is limited in its ability to accurately diagnose MCI and AD by: (i) the effects of cybersickness; (ii) the difficulty of the test, and age-related factors; (iii) the presence of comorbidities; (iv) the use of expensive equipment; (v) the small environment; and (vi) the non-standardization of virtual environments.

### Cybersickness

Proprioception, vision, and vestibular information are constantly being gathered and analyzed by the brain (Commins et al., 2019; Weech et al., 2019). One’s expectation as to how sensory stimuli will be perceived in VR must be congruent with their prior experiences of real-world sensations or else a mismatch may cause the user to experience disorientation, nausea, malaise, and discomfort known as cybersickness (Moffat et al., 2001; Kober et al., 2013; Weech et al., 2019). VR usage may be limited by the participants’ ability to tolerate the experience of cybersickness.

Sense of presence and immersion levels are inversely related to the level of cybersickness experienced (Weech et al., 2019). Evidence suggests that sense of presence can be improved by introducing binaural auditory information and tactile feedback while increasing the frame rate and field of view (Weech et al., 2019). Similarly, physical interaction with a VR, such as by walking in place to move or using handheld devices to manipulate the environment, increases immersion levels and consequently decreases cybersickness (Weech et al., 2019). Increased immersion in future studies may yield stronger test results as participants will be less likely to be lost due to cybersickness.

### Test Difficulty and Age-Related Factors

The difficulty of an assessment was shown to be critically important for discriminating between different degrees of cognitive impairment. A series of VR tests with increasing difficulty showed that MCI patients performed markedly worse than HCs on higher difficulty tests but could not be differentiated from HCs on lower difficulty tests (Lesk et al., 2014). The reliability of most cognitive functioning assessments is likely dependent on difficulty. In addition to surpassing a minimum difficulty threshold, a maximum difficulty is equally important to outline. A hypothetical test, in which the difficulty is too high, would result in HCs being unable to score well enough to differentiate themselves from MCI and AD patients.

A perceived difficulty may worsen spatial memory performance as well (Commins et al., 2019). This includes perceived difficulty while acclimating to the use of the VR headset, as well as to learning the VR task (Commins et al., 2019). However, Commins et al. (2019) argue that perceived difficulty is not the conclusive cause of poorer spatial memory performance. The perceived difficulty was higher in older participants, but spatial memory, in general, was also noted to decrease with age, making it difficult to distinguish one from the other (Commins et al., 2019; Diersch and Wolbers, 2019). The perceived difficulty and worsening spatial memory may place limitations on the use of VR in the elderly population (Diersch and Wolbers, 2019).

### Presence of Comorbidities: Effects of Vision, Cognitive, Psychiatric, and Motor Disorders

The very nature of both immersive and non-immersive VR is largely dependent on the use of vision to navigate virtual situations. Therefore, inclusion criteria for participants in multiple studies included corrected vision (Cushman et al., 2008; Plancher et al., 2012; Kober et al., 2013; Lesk et al., 2014). Plancher et al. (2012) also elected to exclude those with either color blindness or a field of vision less than 46°, due to the qualitative nature of the answers required for some questions. The visual nature of VR thus limits its user base to those with adequate eyesight. This may be a significant limitation given that MCI and AD patients are typically older adults, and therefore more likely to be visually impaired.

VR testing also requires a certain level of cognitive functioning. Sufficient verbal skills, in the language spoken by the facilitators, were necessary to participate in most trials. All tests required the participants to answer questions about the test (Cushman et al., 2008; Weniger et al., 2011; Plancher et al., 2012; Kober et al., 2013; Lesk et al., 2014; Guderian et al., 2015) and one test required participants to use verbal commands to prompt an operator to manipulate the environment in the desired way (Kober et al., 2013). Before engagement with VR assessments, a participant must fully understand the instructions and task they will be asked to execute. To ensure this, two tests required participants to score a minimum of 17 on the MMSE, indicating cognitive functioning somewhere between healthy cognitive function and mild to moderate dementia (Cushman et al., 2008; Kober et al., 2013). Further cognitive decline might render the patient unable to participate in the assessment or benefit from rehabilitation. Furthermore, some tests excluded all those with neurological comorbidities of any kind, limiting their generalizability (Weniger et al., 2011; Lesk et al., 2014).

Although the literature indicates that VR is a powerful tool for spatial memory assessment, care should be taken when evaluating patients with psychiatric disorders. Three studies have found that depressed subjects performed worse on spatial memory VR tasks (Hofmann et al., 2003; Gould et al., 2007; Caffò et al., 2012). While MCI subjects were found to suffer from depression more often than HCs, MCI subjects were still found to have increased spatial deficits when controlling for depression (Caffò et al., 2012). In a VR spatial memory test, an induced state of increased anxiety was similarly shown to decrease spatial memory formation (Zlomuzica et al., 2016). Additionally, certain dissociative states, such as schizophrenia, have also been shown to negatively affect spatial memory (Fajnerová et al., 2014).

The success of these VR assessments also depends on the motivation of participants to complete the tasks. Most of the assessments require special equipment and a significant investment of the participants’ time. If the patient is uninvolved or disinterested in the tasks it can provide a significant obstacle to the validity of the assessments.

Motor disorders could equally prevent participation as the majority of the modalities discussed require the use of manually operated devices. Similarly, an inability of the patient to move their head would preclude the opportunity to use immersive VR headsets.

### Expensive Equipment

The head-mounted devices are an expensive piece of highly specific equipment that is not readily available in all clinical or experimental settings (Commins et al., 2019). With that said, they are becoming more affordable and can be reused by many patients.

### Small Environments

Navigation in small environments may not be comparable to the natural large-scale navigation of the real-world (Learmonth et al., 2008). The inability to physically move around the space appears to negatively affect spatial memory formation, and this is corroborated by the performance of participants on spatial memory assessments by van der Ham et al. (2015) comparing physically walking through a university, with a VR analog of the same task (Learmonth et al., 2008).

### Non-standardized Environments

The variability between assessments makes their comparison and reliability as a whole problematic. Commins et al. (2019) note that for the virtual Morris Water Maze people have used a circular pool within a larger room as the target, while others have used an island with buried treasure as the goal. Beyond this, the size of the goal can be altered to vary the accuracy required to complete the task, the visual cues at the peripheries can be changed, the length of time allowed can be altered and the number of trials can vary (Commins et al., 2019). This variability is not limited to the Morris Water Maze. A more complicated VR task, such as the cycling and shopping combination task would seem to have nearly limitless variability in terms of external stimuli and routes (Mrakic-Sposta et al., 2018). The non-standardization of virtual environments therefore leads to difficulty in replication across studies.

## VR Spatial Memory Test Designs

The emerging interest in VR has led to the creation of a wide variety of testing frameworks. VR spatial memory tests have been organized based on: (i) the output of the test measured; (ii) the memory domain assessed; and (iii) the amount of active participation involved.

### Output Measures Used

In some instances, including those discussed above, apparent improvements in task completion were shown to be largely due to learning effects (Brooks, 1999; Moffat et al., 2001; Hofmann et al., 2003; Weniger et al., 2011; Lesk et al., 2014; Claessen et al., 2016). This type of route learning may be more accurately a measure of procedural memory, which relies on structures of the brain that are largely spared by AD: the basal ganglia and the cerebellum (Hofmann et al., 2003; Vance et al., 2008). The highly repetitive motor action of navigating a virtual environment *via* a touch screen facilitated participants’ ability to learn routes but did not show improvement of spatial memory in neuropsychological assessments (Hofmann et al., 2003).

The designs of most rehabilitation modalities discussed here measured improvement in a single VR task. Lesk et al. (2014) for example, had the participants learn a route, and the participants were evaluated based on their ability to reproduce the same route. To circumvent learning effects, future assessments should incorporate the use of new, unseen VR scenarios to measure improved spatial memory performance. Additional strategies to combat learning effects may take the form of shortened learning time, fewer allowed attempts, or requiring a higher percentage of correct decisions made.

The efficacy of a VR spatial memory rehabilitation program could be quantified by a patients’ improvement on neuropsychological tests. By referring to alternative assessments, some studies were able to demonstrate an improvement in memory over time, on tasks that did not allow for any bias that might be inherent to VR assessments (Caglio et al., 2012; Mrakic-Sposta et al., 2018). The use of any neuropsychological assessment for this purpose would be contingent on its ability to accurately differentiate between HCs, MCI patients, and AD patients.

### Memory Domain Assessed: Allocentric vs. Egocentric Memory

Allocentric and egocentric spatial navigation are the two fundamental divisions of spatial memory assessed by VR. Allocentric memory allows the participant to make assertions about the locations of landmarks and objects independent of the participant’s position, while egocentric memory relies on the participants’ ability to relate where they are in space with the direction they are meant to be traveling (Spiers et al., 2001; Hort et al., 2007).

A common VR assessment of allocentric memory is the Virtual Morris Water Maze, in which the participant is placed in a series of different starting points within a three-dimensional pool and is asked to explore until they locate the hidden exit (Astur et al., 2002; Hort et al., 2007; Commins et al., 2019). By varying the initial orientation of participants each time, the person is reliant on their memory of the relative locations of surrounding landmarks, such as textured walls, to locate the exit. This is in contrast to the egocentric method of using a fixed starting point as a basis to decide subsequent directions to travel. Participants were assessed on the time it took to find the hidden exit and the directness of the route they chose to take (Astur et al., 2002; Hort et al., 2007). An egocentric memory variation of the classic Morris Water Maze, in which the exit is visible and moving, would provide an object in the visual field of participants to orient their movement towards (Commins et al., 2019). The movement of the exit, in particular, may increase the necessity for use of egocentric memory, as this will encourage the participant to constantly be adjusting their movement relative to the world around them.

Another study had participants travel through a town, with no opportunity for deviation from the route, and assessed them on their memory of the relative locations of objects they saw along the way (Plancher et al., 2012). Participants’ allocentric navigation skills were assessed based on the correctness of their assertions as well as the number of objects they could remember (Plancher et al., 2012).

Allocentric memory was also tested using a virtual town (Spiers et al., 2001). First, participants explored the virtual town and then created a map of the area including the locations of eight significant landmark buildings (Spiers et al., 2001). Patients’ scores were based on the relative error in distance between the landmarks on an ideal map, portraying their exact locations, and their own map.

Weniger et al. (2011) measured allocentric memory by utilizing a virtual park, allowing the participant to freely roam until they located a hidden prize. The task was then repeated and the time to completion was measured. Both allocentric and egocentric memory techniques were reported to have been used to solve the task. In the same study, the authors utilized one of the most common VR measurements of purely egocentric memory: maze recall. Different variations of the maze include a simple maze, a garden walkway, and a virtual city. Participants were shown the correct route and then instructed to complete the maze on their own and were assessed on the number of correct turns they made (Moffat et al., 2001; Weniger et al., 2011; Kober et al., 2013; Lesk et al., 2014).

In the study referenced earlier, Plancher et al. (2012) also tested egocentric memory by asking which direction the participant thought they were meant to turn in relation to specific memorable landmarks. This task is similar to the maze, but it does not allow the participant to rely on the immediate experience of traveling the route to answer the question. Instead, it relies solely on the participant’s memory of a specific location and the direction of the route that followed.

Once exploration had occurred, Spiers et al. (2001) also tested egocentric memory by showing pictures of target landmarks and measuring the length of the participants’ path in locating them. Participants were required to use their memory of the surrounding landmarks to locate their desired target from an egocentric standpoint. Scores were calculated by averaging the path lengths taken to get between all target landmarks in a predetermined order.

Using a variety of the above tests, MCI and AD patients can be selectively assessed for their egocentric and allocentric spatial memory. The organization of assessments into these memory domains provides valuable insight into the challenges faced by cognitively impaired patients. By identifying where their specific cognitive capabilities could benefit from support, patients could potentially improve their quality of life. Further investigation into VR assessments of egocentric and allocentric spatial memory may improve the delivery of care to patients living with cognitive impairment.

### Effect of Active Exploration

Plancher et al. (2012) tested the effects of active and passive exploration on the development of spatial memories and found that all participants, including HCs, MCI patients, and AD patients, were more likely to remember allocentric spatial information when actively maneuvering themselves through the virtual environment. Authors postulated that this effect is due to the incorporation of procedural memory and motor brain regions, which are well preserved in AD (Vance et al., 2008), thereby improving the overall memory of the events (Plancher et al., 2012). The implementation of this information into future rehabilitation strategies may help patients improve their spatial memory by leveraging these other systems (Weniger et al., 2011; Plancher et al., 2012; Claessen et al., 2016).

## Conclusion

Data from the studies included in this review indicate that VR modalities could be used as a cognitive screening and diagnostic tool for MCI and AD (Hort et al., 2007; Cushman et al., 2008; Weniger et al., 2011; Plancher et al., 2012; Cogné et al., 2017; Chua et al., 2019). While this review focuses on the ability of VR to detect spatial memory deficits commonly seen in MCI and AD patients, there is evidence supporting its ability to screen for both diseases based on other cognitive domains including learning and memory, perceptual-motor function, and executive function (Chua et al., 2019). Its improved ecological validity, over the current neuropsychological testing, makes VR an exciting new modality that could enhance early diagnosis of MCI and AD (Hort et al., 2007; van der Ham et al., 2015; Chua et al., 2019). Furthermore, findings indicate the possible utility of VR in the rehabilitation of spatial memory deficits (Caglio et al., 2012; Kober et al., 2013; Montana et al., 2019). Rehabilitation findings are limited, however, and evidence suggests that rather than an improvement in true spatial memory, studies have measured a preserved skill forming capacity mimicking spatial memory (Brooks, 1999; Hofmann et al., 2003; Weniger et al., 2011; Lesk et al., 2014; Claessen et al., 2016).

## Author Contributions

MJ, FA, DC, and SA designed the review. MJ and DC compiled and analyzed the literature. MJ wrote the original draft of the manuscript. MJ, DC, SA, and FA revised the manuscript, read and approved the final version to be published. All authors contributed to the article and approved the submitted version.

## Conflict of Interest

The authors declare that the research was conducted in the absence of any commercial or financial relationships that could be construed as a potential conflict of interest.
